# Competency-based SBIRT training for health-care professionals: nursing and social work students

**DOI:** 10.1186/1940-0640-10-S1-A14

**Published:** 2015-02-20

**Authors:** Heather J Gotham, Sarah Knopf-Amelung, Laurie Krom, Pat Stilen, Kendall Kohnle

**Affiliations:** 1 School of Nursing and Health Studies, University of Missouri-Kansas City, Kansas City, MO, 64108, USA

## Background

Most health-care professional training programs lack educational curricula on substance use disorders and strategies for early intervention or referral to treatment. The University of Missouri-Kansas City Screening, Brief Intervention and Referral to Treatment (UMKC-SBIRT) training project educates baccalaureate nursing, advanced practice nursing, and master’s of social work students through didactics threaded throughout coursework; role-plays with classmates and faculty; standardizes patient practice; and offers clinical experience to help students achieve competency.

## Methods

In year one of the training grant, students completed surveys prior to, immediately after, and 30 days after SBIRT training. Surveys covered attitudes and knowledge. Skills were assessed by expert coding of an audiotaped interaction with a standardized patient actor using an SBIRT fidelity scale. Qualitative feedback regarding training experience, knowledge, and attitudes was collected at post-training focus groups.

## Results

Students showed increased knowledge of SBIRT, improved perceptions toward working with patients who use substances, increased comfort in dealing with substance use issues, and progress in developing skills to provide SBIRT interventions.

## Conclusions

Training on SBIRT can be readily implemented into nursing and social work education, improving future health professionals’ perceptions and providing a valuable skill through which they can help patients lead healthier lives.

